# A Few Common Observations on the Treatment of the Grippe

**Published:** 1919-01

**Authors:** 


**A Few Common Observations on the Treatment of the Grippe.** G. LYON. *Presse Médicale*, October 10, 1918.

The author considers that the grippe assumes three forms:

(a) General infection with fever, general fatigue and pains with adynamia.

(b) Thoracic form with pulmonary congestion, pulmonary edema or broncho-pneumonia.

(c) Gastro-intestinal form.

There is no specific medication since no serum or vaccine has been discovered. Before the epidemic of 1889 quinine was the only remedy known against " the fever ". Since then antypirin, aspirin, pyramidon and phenacetin have been employed, all of which have their faults, one being to cause a profuse perspiration and consequent weakening for the patient. Quinine is a corrective as well as a tonic and also antitoxic. In any case only small doses should be administered whether for salts of quinine or antypirin, or aspirin. Sulphate of quinine is the form most used but bromohydrate is far preferable in the author's opinion.

Powder of quinquinina calisaya, which has been proposed instead of quinine, has not been considered an improvement. Caffein can be associated with bromohydrate of quinine with satisfactory results. Undoubtedly the best product to combat asthenia is sulphate of strychnin. The effects are nothing short of wonderful.

Where the patient is so weak that strychnin would have no effect whatever, adrenalin should be given without delay — 5 drops an hour, amounting to a total of 30 to 40 drops a day by the digestive way.

Tincture of aconite, though old fashioned, should be prescribed. It affords great belief both in respiratory troubles and in lessening neuralgia; 20 to 40 drops in thebaic tincture or in a solution where benzoate of soda is an ingredient, such as the following :

| | | |
|---|---|---|
| Tincture of aconite. . . . . . . . . . . . . | 30 | drops |
| Benzoate of soda. . . . . . . . . . . . . | 2 | grammes. |
| Distilled water of Lauro-cerasus. . . . . . | 5 | " |
| Syrup of tolu . . . . . . . . . . . . . . | 20 | " |
| Thebaic syrup . . . . . . . . . . . . . . | 10 | " |
| Distilled water . . . . . . . . . . . . . . | 150 | " |

Alcohol has been very useful in some cases as a preventive, but all patients do not tolerate it well. Under no pretext should the patient be given anything but strict hydriadies — abundant, 3 liters a day, insuring a thorough washing of the intestinal organs. Purgatives are not recommended until the illness is on the decline.

In cases of congestion cupping is a good remedy, also subcutaneous injections of camphorated oil. In occurrences of pulmonary oedema extensive bleeding is desirable (from 400 to 500 grammes at least) together with alternate injections of camphorated oil and sulphate of strychnine associated with sulphate of spartein.

Chloroformed water is indicated in the gastro-intestinal form, for it reduces food intolerance considerably; a few drops of champagne in water may be given also. Purgatives are useful in this form of grippe, either calomel or sulphate of soda. In case of dysentery subcutaneous injections of glucosed serum are indispensable.

